# Mammary adipocyte flow cytometry as a tool to study mammary gland biology

**DOI:** 10.1002/2211-5463.13620

**Published:** 2023-05-12

**Authors:** Geula Hanin, Anne C. Ferguson‐Smith

**Affiliations:** ^1^ Department of Genetics University of Cambridge UK

**Keywords:** cell sorting, cell surface staining, flow cytometry, mammary adipocytes, mammary gland

## Abstract

The mammary gland is a vital exocrine organ that has evolved in mammals to secrete milk and provide nutrition to ensure the growth and survival of the neonate The mouse mammary gland displays extraordinary plasticity each time the female undergoes pregnancy and lactation, including a sophisticated process of tertiary branching and alveologenesis to form a branched epithelial tree and subsequently milk‐producing alveoli. Upon the cessation of lactation, the gland remodels back to a simple ductal architecture via highly regulated involution processes. At the cellular level, the plasticity is characterised by proliferation of mammary cell populations, differentiation and apoptosis, accompanied by major changes in cell function and morphology. The mammary epithelium requires a specific stromal environment to grow, known as the mammary fat pad. Mammary adipocytes are one of the most prominent cell types in the fat pad, but despite their vast proportion in the tissue and their crucial interaction with epithelial cells, their physiology remains largely unknown. Over the past decade, the need to understand the properties and contribution of mammary adipocytes has become more recognised. However, the development of adequate methods and protocols to study this cellular niche is still lagging, partially due to their fragile nature, the difficulty of isolating them, the lack of reliable cell surface markers and the heterogenous environment in this tissue, which differs from other adipocyte depots. Here, we describe a new rapid and simple flow cytometry protocol specifically designed for the analysis and isolation of mouse mammary adipocytes across mammary gland developmental stages.

## Graphical abstract

Mammary adipocytes are an understudied cellular niche with important implications for lactation and endocrine signalling. The dynamic and cellular heterogeneity of this tissue makes it challenging to study. We have established a new efficient and simple flow cytometry protocol specifically designed for the analysis and isolation of mouse mammary adipocytes throughout the developmental cycle of the mammary gland.

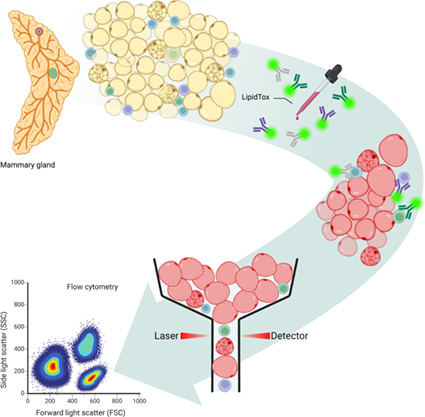

**Abbreviations**
AF488: Alexa Fluor 488
DDW: double distilled water
FBS: fetal bovine serum
HEPES: 4‐(2‐hydroxyethyl)piperazine‐1‐ethanesulfonic acid, *N*‐(2‐hydroxyethyl)piperazine‐*N*′‐(2‐ethanesulfonic acid)
KRB: Krebs‐Ringer modified buffer
PBS: phosphate‐buffered saline

The mammary gland is a unique organ that evolved in mammals to provide postnatal nutrition to the offspring via lactation [1, 2]. Mouse mammary gland development is a highly regulated cyclical process which is repeated for each pregnancy. During puberty, it is manifested by morphological changes, including the formation of terminal end buds, mammary epithelial ductal tree growth and bifurcation. During gestation, due to exposure to hormones such as oestrogen, progesterone and prolactin [3], the mammary epithelium undergoes additional tertiary branching and alveologenesis to form alveoli which will become functional and produce milk to nourish the offspring after birth [1].

The processes of normal mammary gland development require a specific environment, the mammary fat pad, which mostly comprises mammary adipocytes [4, 5]. Research has shown that the fat pad not only provides an appropriate stroma for the development of the epithelial tree but may also serve as an endocrine organ to facilitate adipokine and cytokine signalling, similar to other adipose depots [6].

The nulliparous mammary gland contains mostly white adipocytes. Lactation stimulates mammary adipocytes to change their physical properties and lose most of the lipids which provide an initial source of lipids for milk fat production [4]. During involution, a two‐stage process of programmed cell death and extensive signalling leads to the first stage of involution, characterised by the activation of lysosomal pathway of cell death, known as lysoptosis. The first phase of involution lasts up to 48 h in the mouse and is triggered by milk stasis in the alveolar lumens. Key proteins regulate this phase, including Stat3, JAK1 and LIF. This process is reversible if suckling resumes at this stage. Subsequently, activation of the second irreversible phase of involution occurs, characterised by the drop in circulating prolactin levels [7]. Throughout involution, mammary adipocytes increase in size and expand to repopulate the tissue in a process that is not fully understood.

In recent years, there has been increased awareness of the unique roles of different cell types within this tissue, which present distinct transcriptional profiles, dynamics and mechanobiology [8, 9, 10].

Despite the vast proportion of mammary adipocytes in the tissue, most of the studies in the field focus on the mammary epithelium, while the physiology and molecular mechanisms of mammary adipocytes remain largely unknown. In the last three decades, the function of adipose tissue has become recognised, with growing knowledge revealing the cellular diversity and the roles of different types of adipose tissue, namely white, brown and beige adipose. It is now clear that adipose is not solely a lipid storage depot but also an endocrine and thermogenic organ [11], which includes adipose stromal and stem cells with distinct markers related to the anatomical location and pathophysiology of metabolic diseases [12].

At the end of pregnancy and during lactation, mammary adipocytes show unique characteristics which are not observed in normal white or brown adipocytes. Those include unique morphology, compartmentalised lipid droplets, cytoplasmic septa, large mitochondria, cytoplasmic projections, numerous peroxisome and abundant rough ER. Some researchers suggest that during lactation, mammary adipocytes undergo trans‐differentiation or adipoepithelial conversion and refer to them as pink adipocytes [13, 14]. However, further evidence is required to fully understand the identity of mammary adipocytes during lactation.

New techniques to study adipose were implemented, including immortalised preadipocyte and adipocyte cultures and new mouse models such as Cre‐loxP mediated lineage. However, many available methods are appropriate for a largely homogeneous adipose tissue but have not been not explicitly adapted to the mammary gland.

Exploring mammary adipocytes poses various challenges because these cells reside in a highly plastic environment which changes dramatically with development. For example, mammary adipocytes enlarge during mid‐gestation, change their lipogenic capacity and either deplete or de‐differentiate after parturition [5, 14].

During lactation, their physical properties change dramatically, and their numbers drop, making it more difficult to localise them [15].

In the last few years, several groups successfully isolated fresh adipocytes using flow cytometry [16, 17], overcoming challenges such as their large cell size, fragility and buoyancy. However, this was performed in adipose tissue, a very different cellular context which is not directly transferable to the mammary gland.

Here, we developed a protocol designed to address the specific challenges of sorting mammary adipocytes. We mitigate mammary cellular heterogeneity and adjusted our protocol to fit nulliparous tissue as well as adult developmental stages, including gestation, lactation and involution, allowing single‐cell exploration of mammary adipocytes.

This protocol works efficiently for all adult developmental stages and allows the isolation of over 10 000 cells in all tested stages. Typical numbers of isolated cells that were obtained from the cell sorter are shown in Fig. 4A.

## Materials

### Animal handling and tissue preparation

1. Isoflurane vaporiser or CO_2_ chamber
2. Ethanol 70%
3. Fine sharp tip scissors
4. Lexer‐Baby Scissors, blunt tip
5. Serrated Forceps (Narrow pattern 2 mm tip)
6. Tissue Forceps
7. Cotton swabs
8. Polystyrene surface
9. Aluminium foil
10. Phosphate‐buffered saline pH = 7.4 (PBS ×1) prepared from tablets (Thermo Fisher Scientific, Waltham, MA, USA; BR0014G)
11. Scalpel
12. 20 G needles
13. Petri dish
14. pH meter
15. 0.22 μm filter

### Digestion

1. Collagenase from *Clostridium histolyticum* (Sigma‐Aldrich, Gillingham, UK; C2139)
2. Fetal bovine serum (FBS), heat‐inactivated (Fisher Scientific, Arendalsvägen, Göteborg, Sweden; 11550356)
3. Gentamycin (Sigma‐Aldrich, Gillingham, UK; G1397)
4. Adenosine (Sigma‐Aldrich; A9251)
5. Sodium Chloride, NaCl (Fisher Scientific; 10735921)
6. Potassium Chloride, KCl (Sigma‐Aldrich; P3911)
7. Magnesium Sulfate, MgSO_4_ (Sigma‐Aldrich; M75060)
8. Potassium phosphate monobasic, KH_2_PO_4_ (Sigma‐Aldrich; P5655)
9. Dextrose, d‐(+)‐Glucose (Sigma‐Aldrich; G8270)
10. 4‐(2‐Hydroxyethyl)piperazine‐1‐ethanesulfonic acid, *N*‐(2‐Hydroxyethyl)piperazine‐*N*′‐(2‐ethanesulfonic acid), HEPES (Sigma‐Aldrich; H3375)
11. Calcium Chloride, CaCl_2_ (Sigma‐Aldrich; C1016)
12. 200‐μm mesh PluriStrainer filter (PluriSelect, Leipzig, Germany; 43‐50200‐50)
13. Hanks balanced salt solution without phenol red (Sigma; H6648)
14. Orbital shaker incubator
15. Centrifuge

### Staining and sorting

1. LipidTox Deep Red (Thermo Fischer; H34477)
2. 12 × 75‐mm polypropylene tubes
3. Versene solution (Gibco; 1504033), or 0.2 g·L^−1^ EDTA(Na_4_) in 1× PBS
4. Normal Rat serum (Sigma‐Aldrich; R9759)
5. Antibodies to exclude other populations:
  ○ CD45 biotin (Thermo Fischer; 13‐0451‐85)
  ○ CD31 biotin (Thermo Fischer; 13‐0311‐85)
  ○ CD49f Alexa Fluor 488 (AF488) (BioLegend; 313608)
  ○ Ter119 biotin (Thermo Fischer; 13‐5921‐82)
  ○ BP‐1 biotin (Thermo Fischer; 13‐5891‐81)
6. Alexa Fluor 488 (AF488) Steptavidin (BioLegend, San Diego, CA, USA; 405235)
7. Flow cytometer, BD ARIA III (BD Biosciences, Wokingham, UK)

### Software

flowjo, Ashland, OR, USA. or similar software for flow cytometry analysis.

## Methods

### Solutions preparation

Prepare the following solutions as described below:

1. Krebs‐Ringer Modified Buffer (KRB)

**Table:** 

Reagent	Weight per 1 L (g)	Final concentration (mm)
NaCl	7.008	120
KCl	0.35	4.7
MgSO_4_	0.144	1.2
KH_2_PO_4_	0.163	1.2
Glucose	0.99	5.5
HEPES	2.383	10
CaCl_2_	0.244	2.2
2. Bring the solution to 1 L with double distilled water (DDW). Adjust the pH to 7.4 with NaOH and HCl. Filter through 0.22 μm filter. Store at 4 °C.
3. Digestion buffer
  Prepare 10 mL digestion buffer per animal and filter through a 0.22 μm filter. This buffer can be prepared in a large batch, aliquoted in 15 mL falcon tubes and kept at −20 °C.

**Table:** 

Reagent	Final concentration
Collagenase	1 mg·mL^−1^
FBS	2%
Gentamycin	50 μg·mL^−1^
Adenosine	200 mm
KRB	10 mL

3 Wash buffer

**Table:** 

Reagent	Final concentration
Adenosine	200 μm
FBS	2%
HBSS	Add to 100 mL

4 Blocking buffer

**Table:** 

Reagent	Final concentration
Normal rat serum	10%
HBSS	Add to 1 mL

### Animals

Mouse work and the experiments for this study were approved by the University of Cambridge Animal Welfare and Ethical Review Body and performed under the UK Home Office Animals (Scientific Procedures) Act 1986. (Home Office project licence # PC213320E).

**Fig. 1:**
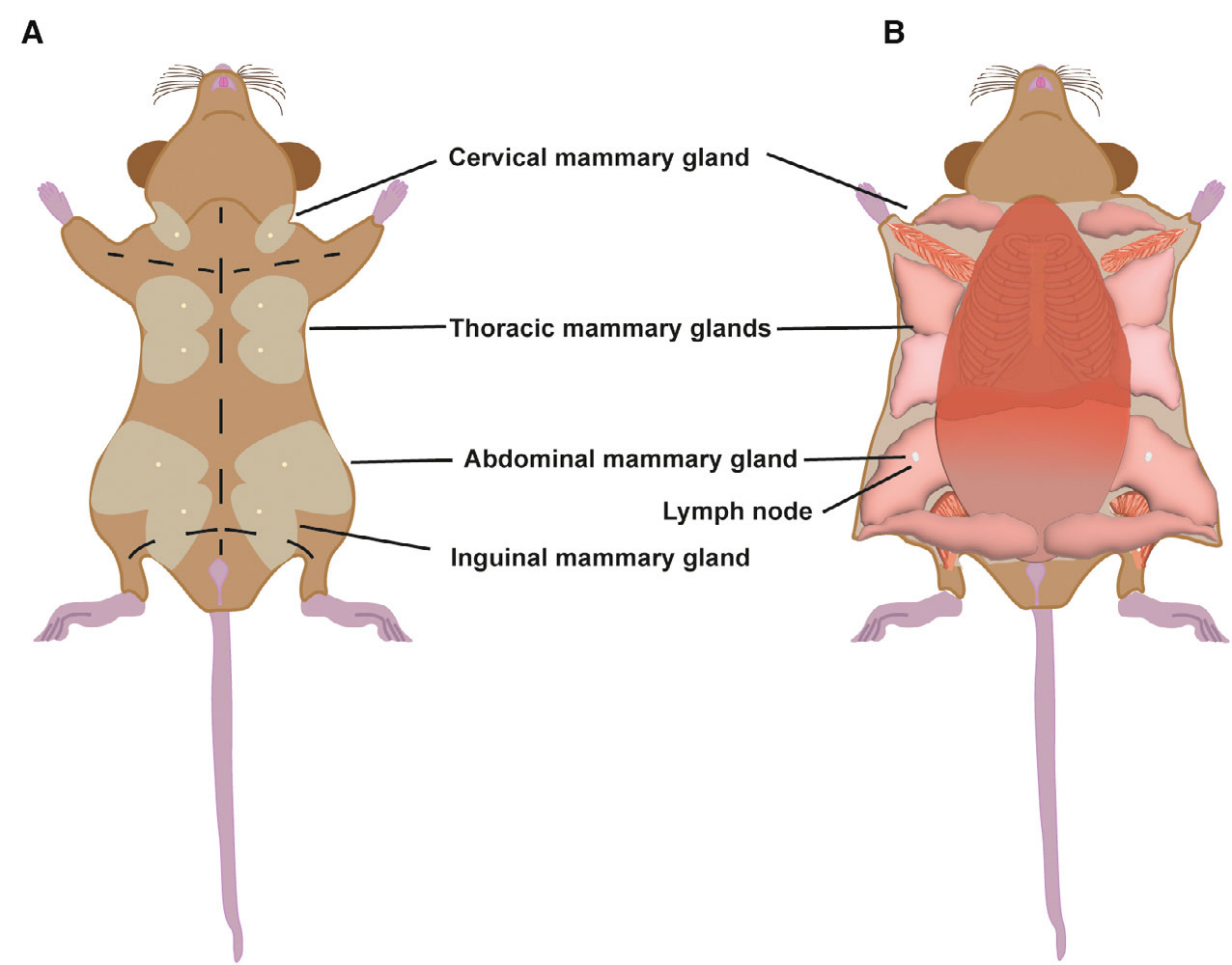
(A) Schematic representation of mouse mammary glands. Black dashed line represents the incisions necessary for the dissection. (B) Open necropsy of mouse mammary glands.

1. Select an appropriate animal and euthanise it using a CO_2_ or isoflurane chamber. If possible, avoid using cervical dislocation as it causes blood accumulation around the cervical mammary glands. For this experiment, use 10–12‐week‐old females (or older) who may be from nulliparous animals, or pregnancy, lactation or involution stages.
2. Place the mouse on its back on a polystyrene surface wrapped with aluminium foil, spread its limbs and pin using 20 G needles.
3. Spray the animal with 70% ethanol.
4. Using serrated forceps, pull the skin above the urethral orifice and make a small incision using fine sharp tip scissors.
5. Using blunt‐tip Lexer‐baby scissors, perform a blunt dissection to gradually separate the peritoneum from the subcutaneous facia and make a medial cranial incision to the jawline.
6. Make four additional incisions from the midline towards the limbs (Fig. 1A).
7. Dip a cotton bud in PBS ×1, slide in the medial incision and roll in both caudal and cranial directions. Perform this bilaterally.
8. Spread the skin and pin to the polystyrene using 20 G needles.
9. Dissect and remove the lymph node from the inguinal mammary gland (#4) using serrated forceps and fine‐tip scissors (Fig. 1B).
10. Dissect mammary gland tissue using fine‐tip scissors and tissue forceps (Fig. 1B).

### Digestion and generation of single cells

**Fig. 2:**
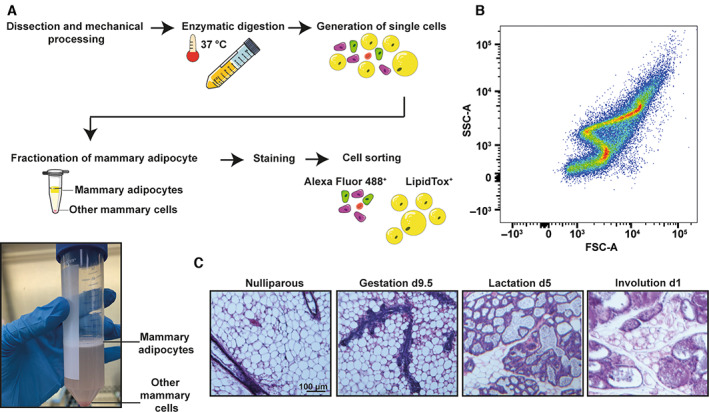
(A) Top: Schematic representation of mammary cell processing from dissection until cell‐sorting stage. Bottom: Image of the adipocyte layer in a falcon tube after spinning. (B) Typical flow cytometry dot plot for a mammary adipocyte sample generated using the described protocol. (C) Representative haematoxylin and eosin stainings of mammary glands from nulliparous, gestation Day 9.5, lactation Day 5 and involution Day 1. This protocol was performed on mammary glands from these time points and additional ones. Scale bar = 100 μm.

1. Place the dissected tissue on ice and add some drops of PBS ×1. When dissecting tissue from lactation or early involution stages, wash the tissue several times in PBS ×1. Mince the tissue using a scalpel and forceps into very fine pieces of approximately 1 mm.
2. Transfer the tissue fragments to a 50‐mL falcon tube with a 10 mL digestion buffer.
3. Incubate the suspension on an orbital shaker incubator for 1 h at 37 °C at 100 r.p.m. and make sure the tube rolls freely on the shaker.
4. Pass through a 200 μm mesh PluriStrainer filter and wash the cells by pipetting 10 mL wash buffer.
5. Centrifuge the filtered suspension at room temperature, 150 ***g***, for 5 min. Following the centrifugation, mammary adipocytes will float and form a supernatant layer.
6. Using a p1000 μL tip, pipette the supernatant mammary adipocytes layer and move into a 15 mL falcon tube.
7. Wash the cells by gently resuspending them in 5 mL wash buffer.
8. Harvest the cells by centrifugation at room temperature, 150 ***g*** for 5 min. Transfer the adipocyte layer to a 1.5 mL tube (Fig. 2A).

### Cell staining

1. Resuspend the adipocytes in blocking buffer and block the sample for 15 min at room temperature.
2. Centrifuge at room temperature, 150 ***g***, for 5 min.
3. Following centrifugation, mammary adipocytes will float. Collect the supernatant layer and add wash buffer to 500 μL.
4. Aliquot your sample into 1.5 mL tubes and spare 10% (50 μL) for negative controls.
  Controls shall include unstained cells and single‐channel staining (in this case, AF488 and LipidTox Deep Red). Add 450 μL wash buffer to each control tube to reach an equal volume for the staining.
*A smaller volume can be spared for negative controls and pooled together from multiple samples when sorting mammary adipocytes from several animals.
5. The rest of the sample (450 μL) will be used for full staining.
6. Add LipidTox Deep Red to the adipocyte suspension at a 1 : 125 dilution (4 μL into 500 μL) and mix gently by inverting the tube.
7. Add fluorescent antibodies of interest (BP‐1‐Bio, Ter119‐Bio, CD45‐Bio, CD31‐Bio, CD49f‐AF488 and add 1 μL of each antibody into 500 μL).
8. Incubate the cells at room temperature for 15 min and mix every 5 min.
9. Centrifuge cell suspension at 150 ***g*** for 5 min. Remove the subnatant from the layer of floating mammary adipocytes.
*At this stage, we remove the subnatant rather than transferring the adipocyte layer (supernatant) between tubes to limit the handling and lysis of the fragile mammary adipocytes.
10. Wash the cells by resuspending the mammary adipocytes in 500 μL wash buffer.
11. Add Streptavidin‐AF488 (1 μL per 500 μL) and incubate for 2–3 min.
12. Centrifuge at 150 ***g*** for 5 min. Remove subnatant.
13. Resuspend cells in 500 μL Versene.
  Keep the sample at room temperature until sorting (Fig. 2A).

### Flow cytometry

**Fig. 3:**
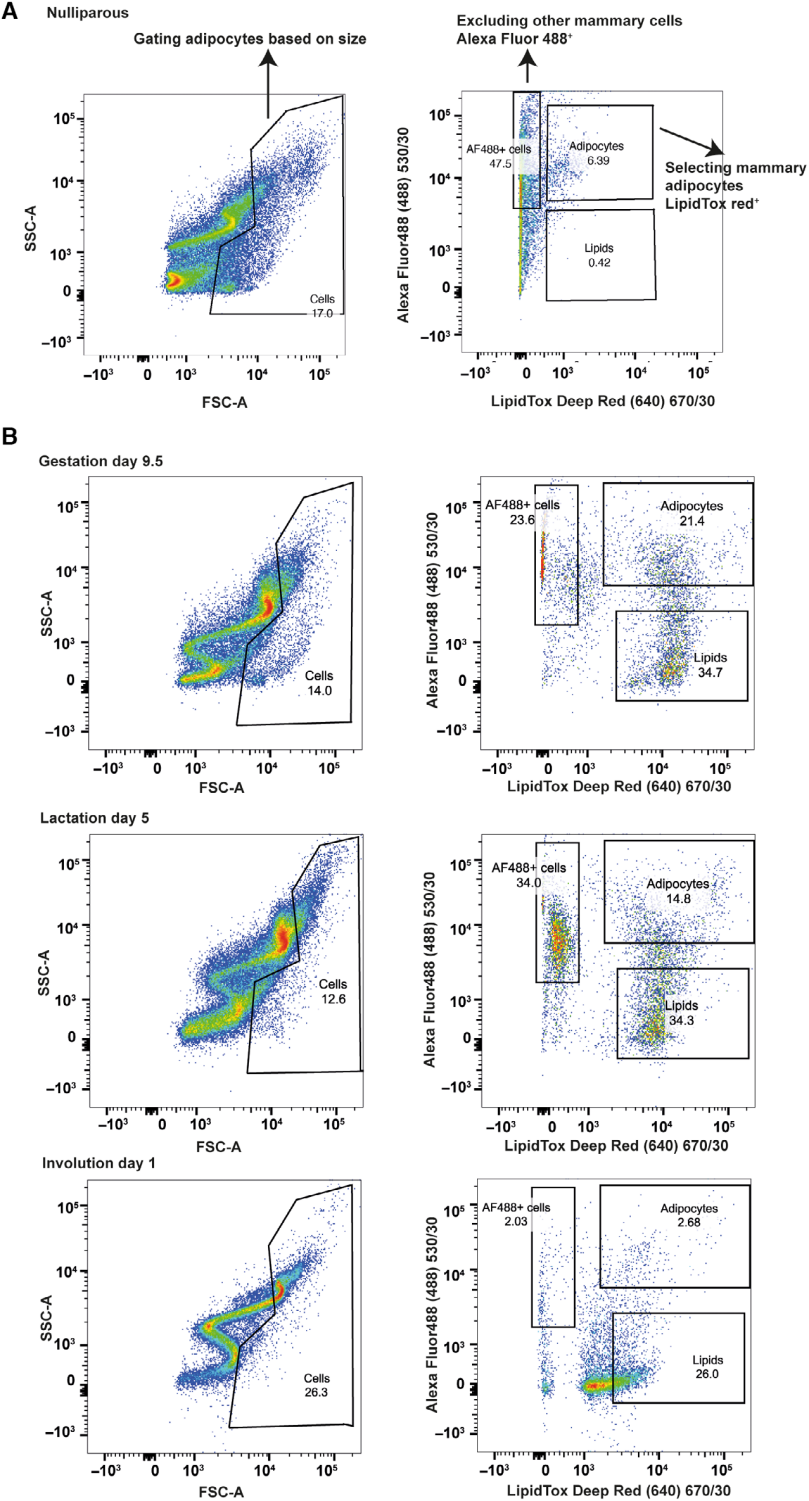
(A) Gating strategy for mammary adipocytes flow cytometry. In the first stage, adipocytes are identified based on their large size using forward and side scatter plots. This population is then divided based on Alexa Fluor 488 labelling CD45, CD31, CD49f, Ter119 and BP‐1, which are used to exclude immune, endothelial and epithelial cells, erythrocytes and tumours, respectively. LipidTox Red fluorescence labels adipocytes and neutral lipid droplets. (B) Density plots of sorted adipocytes at different developmental stages of the mammary gland. Top: Gestation Day 9.5, middle: Lactation Day 5, bottom: Involution Day 1.

1. Start up the flow cytometer in a routine procedure. This procedure may vary for different cell sorters. But would typically include:
  - Cytometer fluidic startup.
  - Start the stream.
  - Set up a breakoff point.
  - Set up a fluidic chart.
  - Validate the performance of the cytometer using beads.
2. Instal the 130 μm nozzle. Some instruments also offer a 150 μm nozzle which may be used.
3. Set up sheath pressure to 10 PSI. High flow pressure may result in the lysis of the cells.
4. Set up the sorting mode to purity.
5. Record your unstained sample and set up gates using single‐channel stained cells (Fig. 2B).
  In this example, samples were sorted using the following excitation and emission parameters:
  Alexa Fluor 488 excitation: 488 nm emission: 450/30 nm.
  LipidTox Deep Red excitation 640 nm emission: 670/30 nm. The gating strategy is detailed in Fig. 3A, using a nulliparous animal.
6. Keep the collection tubes at room temperature and sort the cells into 100 μL wash buffer.

## Tips & Tricks

### Sample selection and processing

- Dissecting mammary glands differs between nulliparous mice and gestation/lactation stages. During gestation and lactation, the gland will become larger and thicker, and blood vessels will be more apparent. During gestation, the glands will become pinker in their colour, and during lactation and early involution, become white due to the presence of milk. This does not affect the way the glands are identified and removed. Representative haematoxylin and eosin‐stained sections of some of the sorted samples show a significant difference in the histology of the tissue (Fig. 2C).
- Identifying the lymph nodes in the inguinal mammary gland (#4) can be challenging during lactation and early involution due to milk engorgement. We recommend practising on nulliparous mammary glands before moving to the latter stages.
- The preparation of the single‐cell suspension is a key factor in the efficiency and reproducibility of this protocol. Reduced digestion time or temperature will result in a lower yield of cells.
- During the digestion, it is important to ensure that the falcon tube containing the tissue and digestion buffer can move freely on the orbital shaker.
- Avoid vortexing the cells, as mammary adipocytes tend to be fragile, and vortexing may result in cell lysis.
- Versene is used to minimise the sticky nature of the sample during the sorting stage at the flow cytometer; however, heparin may be used as an alternative. Using Versene instead of FBS provides a better signal‐to‐noise ratio in flow cytometry.

### Staining and flow cytometry

- This protocol uses antibodies for cell surface Alexa Fluor 488 staining which here labels CD45, CD31, CD49f, Ter119 and BP‐1 that were chosen to exclude immune, endothelial and epithelial cells, erythrocytes and tumours based on well‐established protocols [18]. LipidTox Deep Red fluorescent dye was chosen for labelling adipocytes and neutral lipid droplets based on a previously published flow cytometry protocol for adipose tissue [16]. Further optimisation may be required to explore different subtypes of mammary adipocytes.
- In some flow cytometers, using a neutral density (1.5 ND) filter added in front of the FSC detector improved the detectability of mammary adipocytes. ND filtering allows the detection of large particles, events that may be off the scale on the FSC axis. By decreasing the FSC signal, the events corresponding to mammary adipocytes are kept on the scale.
- In addition to the ND filter, setting up a high FSC threshold allows the detection of larger events. Both options may be used to improve the detection of mammary adipocytes.
- Mammary adipocytes may have very high autofluorescence. Therefore, it is essential to use unstained cells and single‐channel staining to set up the gates. Using fluorescently tagged beads will result in unreliable cell sorting.
- The buoyancy of mammary adipocytes will result in phase separation of the cells and the Versene as soon as the sample is still. As soon as phase separation occurs or a drop in detected events is observed, pause the cell sorting, close the 1.5 mL tube and flick it until the sample becomes homogenous. On average, this is required every 5 min.
- When sorting mammary adipocytes from lactation or early involution stages, the sample may be cloudy due to the presence of milk or milk fat globules in the sample. These events may be gated out as they appear in the lower end of the FSC signal. Additionally, the number of events per second is expected to be very high, and the efficiency would be lower than usual. Figure 3B shows typical flow cytometry plots for gestation Day 9.5, lactation Day 5 and involution Day 1.

### Considerations of cell numbers

**Fig. 4:**
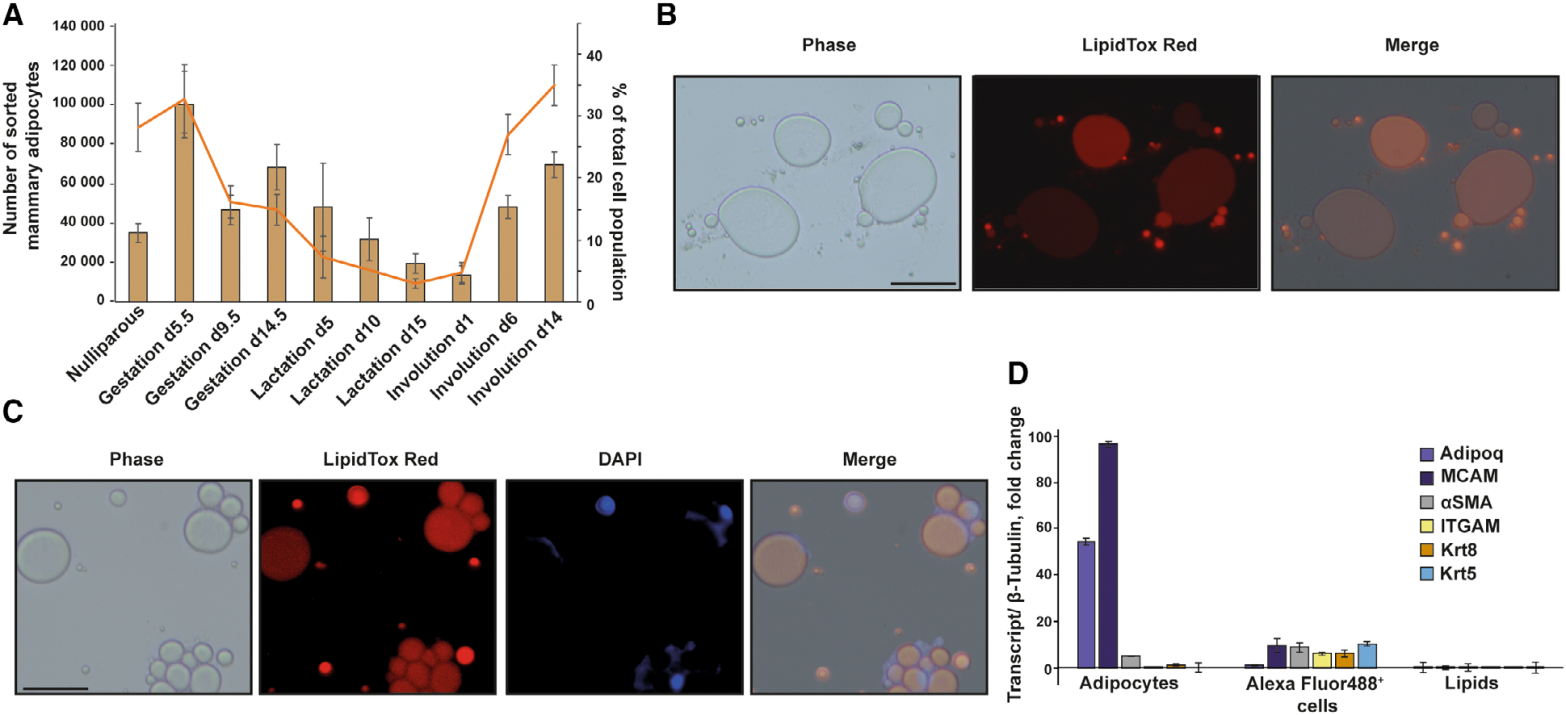
(A) Absolute numbers and percentages of isolated mammary adipocytes. Bars represent the absolute number of sorted mammary adipocytes across mammary gland development (left *y*‐axis). The line represents the percentage of mammary adipocytes of the total number of sorted cells in a single experiment where the pellet was used to isolate epithelial, endothelial and stromal cells. *n* = 8 biological replicates for each time point, error bars represent SEM. (B) Representative images of unsorted mammary adipocytes following the protocol, stained with LipidTox Red, scale bar = 50 μm. (C) Representative images of isolated mammary adipocytes, which were subsequently stained with DAPI, scale bar = 50 μm. (D) Expression profiles of isolated populations of mammary adipocytes, Alexa Fluor488^+^ cells and lipids, based on the gating strategy described in Fig. 3. Expression profiles were quantified using quantitative real‐time PCR. *n* = 4 biological replicates, error bars represent SEM.

- Throughout the development of the mammary gland, the tissue undergoes substantial morphological changes, affecting the size of the tissue and the number of cells. Specifically, mammary adipocytes make up a significantly smaller proportion of mammary tissue during gestation and lactation, when milk‐producing alveoli populate the tissue. During involution, mammary adipocytes become visible and gradually contribute more to the cellular composition of the gland. These changes affect the number of sorted mammary adipocytes at those developmental stages and the efficiency of the cell‐sorting process. Since the tissue becomes larger during gestation and lactation and remains relatively large at early involution, the number of sorted adipocytes from all glands is at least 10 000 cells, even when fewer mammary adipocytes are visible in the tissue. Figure 4A shows the number of sorted mammary adipocytes for each time point.

### Validation of sorted populations

- It is highly advisable to use several independent validations for this protocol:
  ○ Use fluorescent microscopy to visualise the stained cells before and after sorting them (Fig. 4B,C, respectively). You may wish to add DAPI to a small aliquot of the sorted cells to ensure that you have sorted cells rather than lipid droplets (Fig. 4C).
  ○ Extract RNA and perform qPCR validations on the sorted fractions, including negative cell populations, to ensure that the sorted mammary adipocytes express adequate markers, such as Adipoq, and are not expressing epithelial markers, such as Krt5. Figure 4D shows a typical expression profile of the different populations, demonstrating relevant markers highly expressed in sorted mammary adipocytes. Adiponectin was expressed 54‐fold higher in adipocytes compared with Alexa Fluor 488^+^ cells, while MCAM, also known as CD146, was expressed 10‐fold higher. We suggest the following primer pairs in Table 1 for qPCR validation.

**Table 1:** Primer sequences.

Cell type	Primer	Forward sequence	Reverse sequence
Basal	Krt5	TCTGCCATCACCCCATCTGT	CCTCCGCCAGAACTGTAGGA
Luminal	Krt8	ACTCACTAGCCCTGGCTTCA	TCTTCACAACCACAGCCTTG
Stromal	αSMA (Acta2)	GTCCACCTTCCAGCAGATGT	TTGTCGATTGTCGTCCTGAG
Adipocytes and endothelial	MCAM	CCCAAACTGGTGTGCGTCTT	GGAAAATCAGTATCTGCCTCTCC
Immune	ITGAM	ATGGACGCTGATGGCAATACC	TCCCCATTCACGTCTCCCA
Adipocyte	Adipoq	TGTTCCTCTTAATCCTGCCCA	CCAACCTGCACAAGTTCCCTT
N/A (housekeeping gene)	β‐Tubulin (Tubb)	TTCAGCTGACCCACTCACTG	AGACAGGGTGGCATTGTAGG

### Advantages and limitations

The current protocol provides an efficient way to isolate mammary adipocytes. To date, adipocyte cell sorting is not a common practice in this field due to technical difficulties, the physical properties of these cells and non‐standard cell‐sorting parameters. Existing protocols to isolate adipocytes were developed for adipose tissue rather than for mammary glands, which differ substantially and present a challenging cellular heterogeneity.

This unique protocol has been developed specifically for the mammary gland. It addresses the challenges this tissue presents, including isolating a relatively small population of mammary adipocytes during lactation and involution.

The experimental design proposed in this protocol can be further optimised for culturing mammary adipocytes following cell sorting. This will require the addition of live/dead staining, a standard practice when culturing isolated cells. In our hands, live/dead staining such as DRAQ5 or propidium iodide caused artefacts and autofluorescence, which compromised the compensation in the cell sorter. Therefore, we chose not to use live/dead staining. Further adaptations of this protocol will be required for subsequent cell culturing.

Further optimisations may be required to characterise the isolated population of mature adipocytes in flow cytometry, for example using cell surface markers such as CD36, a marker of preadipocytes and mature adipocytes and Sca‐1/CD34, a marker of stem cell niche.

## Conflict of interest

The authors declare no conflict of interest.

### Author contributions

GH conceived and designed the protocol, wrote the paper and made the figures. ACF‐S contributed to writing and edited the paper.

## Data Availability

The data that support the findings of this study are available from the corresponding author ghl35@cam.ac.uk upon reasonable request.

## References

[1] Watson CJ and Khaled WT (2020) Mammary development in the embryo and adult: new insights into the journey of morphogenesis and commitment. Development 147, dev169862. 33191272 10.1242/dev.169862

[2] Slepicka PF , Somasundara AVH and dos Santos CO (2021) The molecular basis of mammary gland development and epithelial differentiation. Semin Cell Dev Biol 114, 93–112. 33082117 10.1016/j.semcdb.2020.09.014 PMC8052380

[3] Brisken C and O'Malley B (2010) Hormone action in the mammary gland. Cold Spring Harb Perspect Biol 2, 1–15. 10.1101/cshperspect.a003178 PMC2982168 20739412

[4] Zwick RK , Rudolph MC , Shook BA , Holtrup B , Roth E , Lei V , van Keymeulen A , Seewaldt V , Kwei S , Wysolmerski J *et al*. (2018) Adipocyte hypertrophy and lipid dynamics underlie mammary gland remodeling after lactation. Nat Commun 9, 3592. 30181538 10.1038/s41467-018-05911-0 PMC6123393

[5] Hovey RC and Aimo L (2010) Diverse and active roles for adipocytes during mammary gland growth and function. J Mammary Gland Biol Neoplasia 15, 279–290. 20717712 10.1007/s10911-010-9187-8 PMC2941079

[6] Ahima RS (2006) Adipose tissue as an endocrine organ. Obesity (Silver Spring) 14 (Suppl 5), 242S–249S. 17021375 10.1038/oby.2006.317

[7] Jeong J , Kim W , Hens J , Dann P , Schedin P , Friedman PA and Wysolmerski JJ (2019) NHERF1 is required for localisation of PMCA2 and suppression of early involution in the female lactating mammary gland. Endocrinology 160, 1797–1810. 31087002 10.1210/en.2019-00230 PMC6619491

[8] Bach K , Pensa S , Grzelak M , Hadfield J , Adams DJ , Marioni JC and Khaled WT (2017) Differentiation dynamics of mammary epithelial cells revealed by single‐cell RNA sequencing. Nat Commun 8, 2128. 29225342 10.1038/s41467-017-02001-5 PMC5723634

[9] Hanin G and Ferguson‐smith AC (2020) The evolution of genomic imprinting: epigenetic control of mammary gland development and postnatal resource control. Wiley Interdiscip Rev Syst Biol Med 12, e1476. 31877240 10.1002/wsbm.1476

[10] Stewart TA , Hughes K , Stevenson AJ , Marino N , Ju AL , Morehead M and Davis FM (2021) Mammary mechanobiology – investigating roles for mechanically activated ion channels in lactation and involution. J Cell Sci 134, jcs248849. 33262312 10.1242/jcs.248849

[11] Giralt M and Villarroya F (2013) White, brown, beige/brite: different adipose cells for different functions? Endocrinology 154, 2992–3000. 23782940 10.1210/en.2013-1403

[12] Milan G , Conci S , Sanna M , Favaretto F , Bettini S and Vettor R (2021) ASCs and their role in obesity and metabolic diseases. Trends Endocrinol Metab 32, 994–1006. 34625375 10.1016/j.tem.2021.09.001

[13] Cinti S (2018) Pink adipocytes. Trends Endocrinol Metab 29, 651–666. 30017740 10.1016/j.tem.2018.05.007

[14] Wang QA , Song A , Chen W , Schwalie PC , Zhang F , Vishvanath L , Jiang L , Ye R , Shao M , Tao C *et al*. (2018) Reversible de‐differentiation of mature white adipocytes into preadipocyte‐like precursors during lactation. Cell Metab 28, 282–288.e3. 29909970 10.1016/j.cmet.2018.05.022 PMC6535147

[15] Colleluori G , Perugini J , Barbatelli G and Cinti S (2021) Mammary gland adipocytes in lactation cycle, obesity and breast cancer. Rev Endocr Metab Disord 22, 241–255. 33751362 10.1007/s11154-021-09633-5 PMC8087566

[16] Hagberg CE , Li Q , Kutschke M , Bhowmick D , Kiss E , Shabalina IG , Harms MJ , Shilkova O , Kozina V , Nedergaard J *et al*. (2018) Flow cytometry of mouse and human adipocytes for the analysis of browning and cellular heterogeneity. Cell Rep 24, 2746–2756.e5. 30184507 10.1016/j.celrep.2018.08.006 PMC6137819

[17] Majka SM , Miller HL , Helm KM , Acosta AS , Childs CR , Kong R and Klemm DJ (2014) Analysis and isolation of adipocytes by flow cytometry. Methods Enzymol 537, 281–296. 24480352 10.1016/B978-0-12-411619-1.00015-X PMC4143162

[18] Prater MD , Petit V , Alasdair Russell I , Giraddi RR , Shehata M , Menon S , Schulte R , Kalajzic I , Rath N , Olson MF *et al*. (2014) Mammary stem cells have myoepithelial cell properties. Nat Cell Biol 16, 942–950. 25173976 10.1038/ncb3025 PMC4183554

